# The Central Asian Supercourse to Increase Scientific Productivity

**DOI:** 10.5195/cajgh.2013.46

**Published:** 2013-03-25

**Authors:** Faina Linkov, Robert Guzman, Sean Soisson, Kyle E. Freese, Aamir H. Sheikh, Eugene Shubnikov, Ronald E. LaPorte

**Affiliations:** 1 Magee-Women’s Research Institute, University of Pittsburgh; 2 iSchool, University of Pittsburgh; 3 Gynecologic Oncology, Magee-Women’s Hospital; 4 Dietrich School of Arts and Sciences, University of Pittsburgh; 5 Institute of Internal Medicine, Novosibirsk, Russia; 6 Graduate School of Public Health, Department of Epidemiology, University of Pittsburgh

**Keywords:** Supercourse, Central Asia, CAREN, Mobile Global Health

## Introduction to Central Asia

The Central Asian countries of Uzbekistan, Turkmenistan, Kazakhstan, Tajikistan and Kyrgyzstan are tied together with similar histories and genetic heritage. Historically, these countries formed from nomadic populations along the Silk Road with common cultures, religions, and languages. They also have similar health patterns and life expectancies (Uzbekistan, 67.2, Kazakhstan, 67.0, Tajikistan, 66.7, Kyrgyzstan, 65.9 and Turkmenistan, 63.2) which are near to the global average life expectancy. 1

Since World War II, Central Asian countries have demonstrated a rapid increase in life expectancy, although there is still a large gap between genders. 2 Each country underwent the epidemiological transition, where life expectancy rose due to a major reduction of infectious diseases and an unmasking of chronic diseases including: diabetes, 3 cardiovascular disease, cancer, stroke, COPD, and injuries. Mortality patterns are virtually the same across Central Asia 4 with variations attributed mainly to differences in lifestyles. 5

## Research in Central Asia

Overall, these countries have had little research collaboration despite sharing similar geographic locations environments, and migration patterns. Central Asian scientific productivity was determined by examining the publication rates for the five Central Asian countries (Table 1). Every country is well below the average worldwide publication rate of countries across the world. Countries such as Ukraine (ranked 38 among all UN recognized countries), Malaysia (43), and Kenya (65) have substantially higher productivity than Central Asian countries. 6

**Figure 2:**
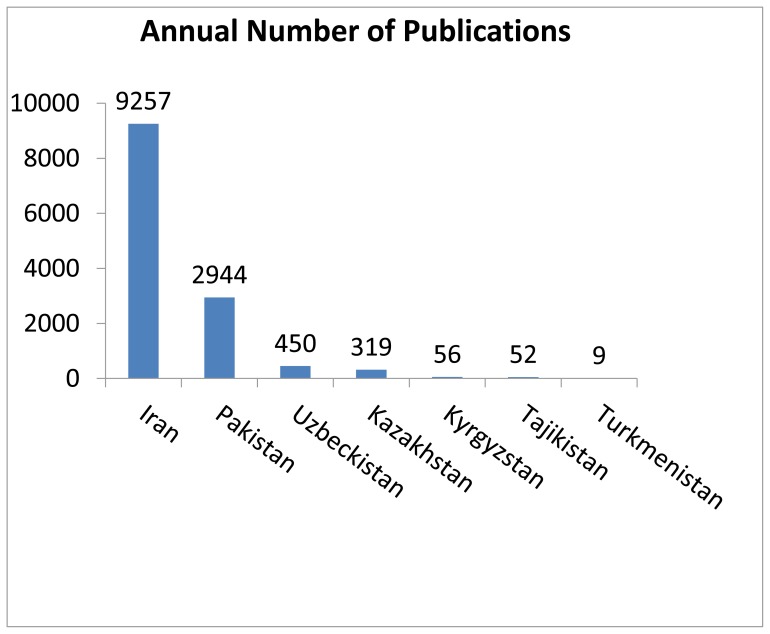
Comparison of number of publications from countries adjacent to Central Asia and the countries of Central Asia. 6

The publication numbers presented in Table 1 represent data from 1993–2010. There are only about 450 publications per year from Uzbekistan and only nine per year from the entire country of Turkmenistan. The five Central Asian countries average only 180 articles per year. In contrast, the annual rate of publication in the U.S. is 409,430 per year, over 2,200 times greater than the average publications per year in each of the Central Asian countries. As presented in Figure 2, there are countries that are adjacent to Central Asian countries, such as Iran, with 40 times the number of articles published (9,257 per year) and Pakistan with 11 times more, 2,944 per year). 6

The scientific disciplines of these publications are similar across these countries. The majority are in physics, chemistry, mathematics, and to a lesser extent, agriculture. There is a paucity of publications in medicine, public health, and social sciences. Clearly, it is important to boost the scientific culture of these countries, especially in the little represented areas.

## Scientific Hope

**Figure 3:**
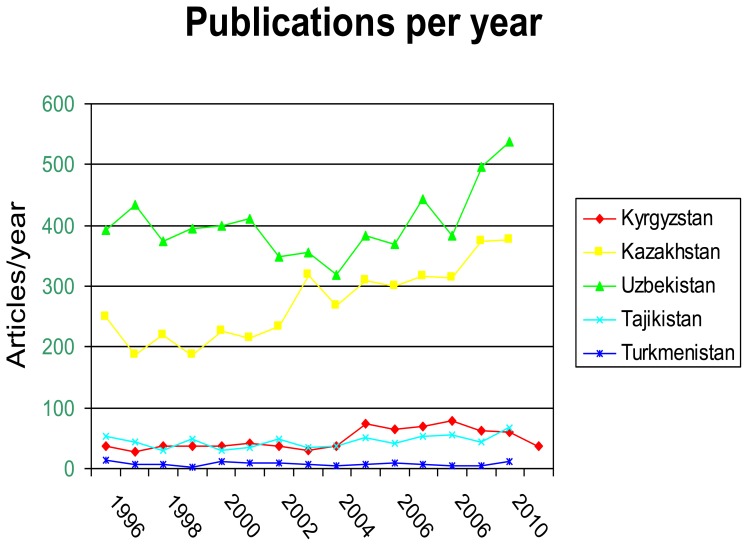
Articles published in the Central Asian countries by year. 6

**Figure 4:**
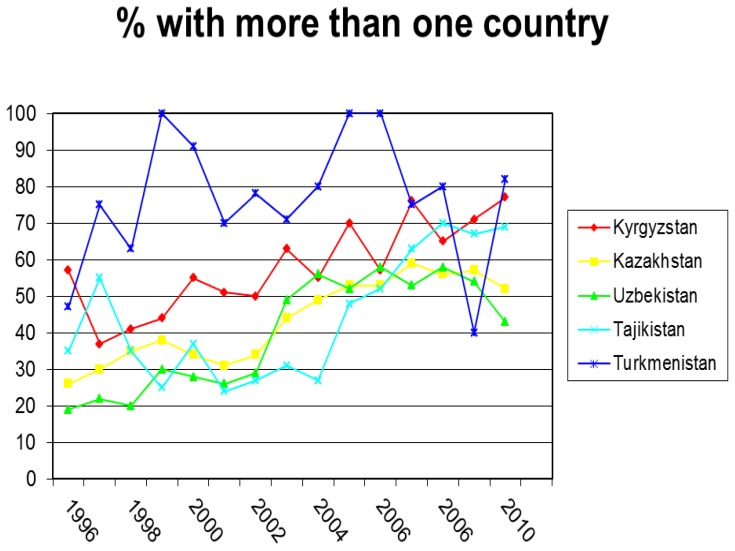
The percentage of articles written in the Central Asian countries which are written with a co-author from another country. 6

Despite the rates of publications being low, there has been a significant increase since 1993, as shown in Figure 3. In Uzbekistan and Kazakhstan, the rise has been substantial, with close to a 100% increase in the number of publications. Moreover, scientists in all five countries have begun collaborating with each other in other countries in the region, as seen in Figure 4. This has nearly doubled international collaboration for all countries in the region. Additionally, over 50% of the articles produced have authors from more than one country. This clearly demonstrates that there is an interest and desire for regional collaboration. 6

The general conclusion is that rates of scientific productivity are relatively low in Central Asia and that focus has been almost exclusively on the “hard sciences” with little global health and behavioral research. However, we are seeing a rise in scientific productivity as well as an interest in international collaboration.

## The Supercourse Team’s Role

Our mission is to build capacity to improve scientific productivity in Central Asian countries. We hope to double the number of publications coming from the region in the next five years through building a global network of scientists, fostering research training, and providing access to broadband connectivity for scientific communication and the sharing of knowledge.

The first element required to improve scientific productivity is to provide networking for the universities in this region. This will lead to the free flow of ideas within and across borders. Secondly, it it is necessary to build a system that will provide training in research. This will be accomplished by creating programs and bolstering classroom research training by creating a Central Asian Supercourse. Finally a human scientific network in these countries will enable people to learn from each other within their countries, the region, and with the world.

## CAREN: (Central Asian Research and Education Network)

The implementation of fiber optic networking for universities in the region will facilitate collaboration between investigators at multiple institutions and will improve research capacity. Launched in January 2009, the Central Asian Research and Education Network (CAREN) is a high-capacity regional research and education network that provides high-speed internet for universities and research centers. The CAREN project, which evolved from the Silk Project funded by the European Commission (EC), is nearing completion. The goal of this project is to improve research collaboration within the region and worldwide. This fiber optic backbone provides the superhighway for research and training programs. 7

To date, CAREN has networked over one-million users in more than 200 universities and facilitates joint regional and global projects. The user communities for CAREN include: distance learning, environmental studies, seismology, telemedicine, and textile research. The CAREN project will next link Central Asia and Europe to connect researchers across the world, increasing the collaboration and fostering development. 4

## The Central Asian Supercourse

Supercourse is a unique combination of a human network and a digital network containing state of the art content about global health and prevention The Supercourse model is very simple. The Central Asian Scientific Supercourse team has built a global network of over 50,000 primarily academic faculty members from 174 countries. The network contains 4855 PowerPoint lectures including: 77 from Nobel Prize laureates, 300 from IOM/NAS members, several lectures from the past two directors of the NIH and CDC, and the US Surgeon general. On average, each of the authors in the Supercourse has published 100 articles. 8

These lectures can be distributed to faculty across the world, and can be used to compile cohesive lectures and presentations from disparate sources. For example, and educator using Supercourse might select five slides from Peter Bennett, an expert on diabetes, eight from their own lectures, and ten from scientists in Europe. Through the Supercourse, teachers are empowered by having access to current, state-of-the-art lecture material.

There are 31 different languages represented in the Supercourse, with over 200 lectures in Russian, and several in Uzbek and Turkmen. The Central Asian Journal of Global Health (cajgh.pitt.edu) is a product of extensive scientific networking and capacity of the Supercourse Project.

## Utilization of Supercourse in Central Asia

Scientists from Central Asia are already participating in the Supercourse. Over 60 faculty members from the region are involved (200 accessing the site within the past 12 months), with most of the lectures from within the region coming from Kazakhstan. In addition, all of the medical schools in the region are connected with the Supercourse and to each other via CAREN.

Supercourse is a two-way street on which scientists in Central Asia and around the globe share their knowledge. We have already seen the benefits of this exchange through the 46 outstanding lectures prepared by participating faculty. These lectures were of interest to the world as they received 913 page views from outside the region, primarily from Europe and the U.S.

Clearly, there is an interest in building research capacity as well as in sharing knowledge within the region. The Central Asian Supercourse facilitates network fulfills this need. The origins of the Central Asian Supercourse can be found at: http://www.pitt.edu/~super1/faculty/centralasia.htm. Its growth will be monitored as it becomes connected to the CAREN network.

## Mobile Global Health

**Figure 5:**
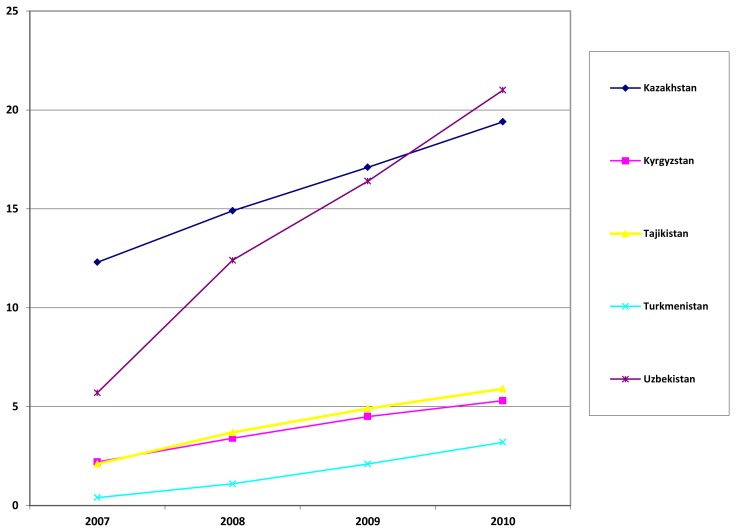
The number of cell phone subscriptions (in millions) for each of the Central Asian countries. 10

The field of “Mobile Global Health” represents the applications of cellular telephone technology to global health and prevention. It is expected that the number of cell phones will be equal to the number of people on earth in the next five years, which means that the vast majority of the global population will be accessible via cell phone. There are especially exciting implications for this trend in Central Asia, where cell phone use is already rising as shown in figure 5. 9,10

Furthermore, Supercourse developer Eric Marler has been capturing all the phone applications pertaining to science. 11 In just the last few months, nine applications have appeared from Central Asia; however, none of these are for science, and instead are focused on the travel industry.

## Conclusion

The Central Asian countries of Uzbekistan, Turkmenistan, Kazakhstan, Tajikistan and Kyrgyzstan are primed for an increase in scientific productivity, measured by anticipated increase in peer reviewed scientific publications. Since the use of cellular/mobile devices is increasing in Central Asia, we support establishing a new field called “Mobile Global Health”. With the help of the CAREN project and our Central Asian Supercourse Team, the region will be able to utilize computer and human networks in order to share scientific research and reach out to each other and around the world.

## Figures and Tables

**Figure 1:**
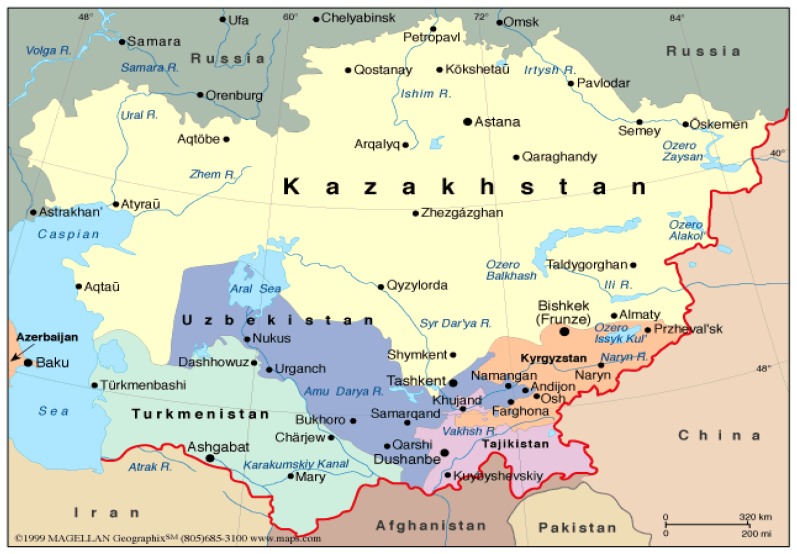
The countries of Central Asia

**Table 1:** The rate of publication for the Central Asian countries and their ranking worldwide. 6

Country	Ranking	Number of scientific publications
Uzbekistan	80	6021
Kazakhastan	91	4153
Krygyzstan	141	735
Tajikistan	145	676
Turkmenistan	186	123
